# Heterologous Overexpression of Cytochrome P450BM3 from *Bacillus megaterium* and Its Role in Gossypol Reduction

**DOI:** 10.3390/toxins17050253

**Published:** 2025-05-20

**Authors:** Wenpeng Fan, Jingjing Cui, Tongxiang Xu, Shiheng Xu, Zulibina Ainiwaer, Qiyu Luo, Caidie Wang

**Affiliations:** Xinjiang Key Laboratory of Herbivore Nutrition for Meat & Milk, Research Center for Biofeed and Animal Gut Health, College of Animal Sciences, Xinjiang Agricultural University, Urumqi 830052, China; fwp13779868871@163.com (W.F.);

**Keywords:** cytochrome P450BM3, heterologous overexpression, gossypol, reduction pathways

## Abstract

Gossypol is a polyphenolic toxic compound present in cotton plants. To determine whether the candidate cytochrome P450BM3 enzymes could reduce gossypol in vitro, functional recombinant cytochrome P450BM3 enzymes were successfully expressed in *E. coli*. Site-directed mutagenesis generated mutants (R162H, R162K, Q129H, Q129N) to explore structural determinants of catalytic efficiency. Both wild-type P450BM3 and mutants exhibited significant ability to reduce gossypol levels, with R162H and R162K showing 33.4% and 24.2% reduced catalytic efficiency compared with the wild-type enzyme, respectively. Q129H and Q129N mutants maintained comparable catalytic efficiency to the wild type. Metabolomic profiling revealed two distinct reducing pathways catalyzed by wild-type P450BM3 and its mutants (R162H/Q129H), involving decarboxylation, hydroxylation, and C-C bond cleavage. This study demonstrated the feasibility of P450BM3 as a highly efficient biocatalyst for reducing gossypol levels, speculated that Arg162 might be a critical active residue, and hypothesized the potential pathways by which P450BM3 catalyzes the reduction of gossypol content, thereby providing a theoretical foundation for the enzymatic reduction of gossypol.

## 1. Introduction

Gossypol is a toxic phenolic compound present in cotton plants in two forms (free gossypol and bound gossypol) [1]. Gossypol exists in two enantiomeric forms, (+) and (−), with the (−)-gossypol considered more active than the (+)-gossypol as an antifertility agent [2,3]. Studies of gossypol have focused on a range of biological activities, including antiviral [4], antiparasitic [5], and anticancer activities [6]. In the feed industry, the presence of gossypol restricts the use of cotton byproducts in animal feed. However, excess intake of free gossypol can depress animal growth and feed efficiency [7], reduce the fertility of bulls [8], and compromise gamete viability in cattle [9]. Additionally, the toxic effect of free gossypol (FG) on non-ruminants is remarkably more potent than that on ruminants [10]. Due to these toxic effects, the use of cotton byproducts in animal feed is significantly restricted. Therefore, the detoxification of gossypol is needed. Current research on the enzymatic degradation of gossypol remains limited, with proposed degradation pathways showing significant variability [11,12,13,14].

Cytochrome P450 monooxygenases (P450s or CYPs) are ubiquitous heme-containing enzymes, which are capable of oxidizing a vast number of molecules. The P450s play an essential role in the assimilation of carbon sources, in secondary metabolism, and in the degradation of xenobiotics [15]. The cytochrome P450BM3 subfamily belongs to a group of so-called self-sufficient P450 monooxygenases. In this group, the complete electron transfer chain and the P450 monooxygenase are expressed as one polypeptide of approximately 119 kDa, the electron transfer system is composed of a flavin mononucleotide (oxidized) (FMN)-containing flavodoxin-type domain and a flavin adenine dinucleotide (FAD)-containing oxidoreductase domain [16]. Saturated and unsaturated medium to long-chain fatty acids (C12–C22) and fatty acid derivatives are good substrates of the cytochrome P450BM3 family members [17]. CYP9A12 from *Helicoverpa armigera* exhibits gossypol-degrading activity [12]. From the structural analysis of the enzyme protein, cytochrome P450BM3 demonstrates inherent structural advantages over other eukaryotic cytochrome P450 enzymes [18]. Previous studies on cytochrome P450BM3 and its mutants in catalyzing aromatic compounds have confirmed their biological functions in aromatic compound catalysis [19,20]. Gossypol, a natural naphthalene aldehyde polyphenolic compound, is characterized by a rigid binaphthalene skeleton and multiple reactive functional groups (e.g., aldehyde and hydroxyl groups), exhibiting typical characteristics of aromatic compounds. Although catalytic degradation of gossypol by cytochrome P450BM3 has not been reported, we speculate that cytochrome P450BM3 possesses the potential to catalyze reactions with gossypol. Our previous research on liquid fermentation using *B. megaterium* with gossypol acetate as the substrate demonstrated a significant upregulation of the *P450BM3* gene [21]. Thus, this study hypothesizes the role of *B. megaterium* P450BM3 in gossypol reduction and aims to heterologously express the P450BM3 gene in *E. coli*, validate its in vitro catalytic effect on gossypol, optimize enzymatic reaction conditions, and employ site-directed mutagenesis to elucidate critical active sites and propose potential gossypol reduction pathways.

## 2. Results

### 2.1. Heterologous Expression of Cytochrome P450BM3 and Optimization of Gossypol Degradation Conditions

#### 2.1.1. Construction of Cloning and Expression Vectors

**Figure 1:**
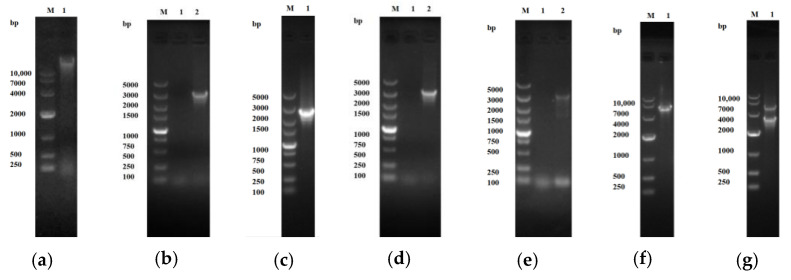
Electrophoretic analysis of cloning and expression vector construction: (**a**) Genomic DNA extraction. Lane M, DL10,000 DNA Marker, lane 1, genomic DNA of *Bacillus megaterium*. (**b**) P450BM3 PCR amplification. Lane M, DL5000 DNA Marker, lane 1, negative control, lane 2, P450BM3 PCR product. (**c**) Linearized cloning Vector pMD19-T. Lane M, DL5000 DNA Marker; lane 1, pMD19-T vector digested with restriction enzymes. (**d**) Recombinant plasmid pMD19-P450BM3 Verification. Lane M, DL5000 DNA Marker; lane 1, PCR product from pMD19-P450BM3. (**e**) Seamless cloning PCR for P450BM3. Lane M, DL5000 DNA Marker; lane 1, negative control, lane 2, seamless cloning product. (**f**) Linearized expression Vector pET28a. Lane M, DL10,000 DNA Marker; lane 1, pET28a vector linearized by restriction digestion. (**g**) Restriction enzyme digestion of pET28a-P450BM3, Lane M, DL10,000 DNA Marker; lane 1, digested pET28a-P450BM3.

As depicted in Figure 1a–c, the genomic DNA of *Bacillus megaterium* ATCC 14581 had a size exceeding 10 kb. The PCR-amplified target gene fragment was approximately 3 kb, which was in accordance with the expected size. Meanwhile, the linearized pMD19-T vector fragment was measured to be around 2.6 kb. PCR analysis of the recombinant clone plasmid pMD19-P450BM3 showed the successful insertion of a fragment approximately 3 kb in size (Figure 1d). Sequencing results indicate that the ligated product, with a length of 3150 bp, matched the sequence length recorded in GenBank. For the PCR seamless cloning process, the target gene fragment was approximately 3 kb, consistent with the expectation (Figure 1e), and the linearized PET28a vector was about 5 kb in size (Figure 1f). The recombinant plasmid PET28a-P450BM3 was identified through double enzyme digestion. A target fragment of approximately 3 kb and a vector fragment of around 5 kb were obtained, both of which were in line with the anticipated results (Figure 1g). Sequencing results demonstrate that the 3150 bp connection fragment was the desired target gene fragment and was capable of being induced for expression.

#### 2.1.2. Optimization of Cytochrome P450BM3 Enzyme Expression

**Figure 2:**
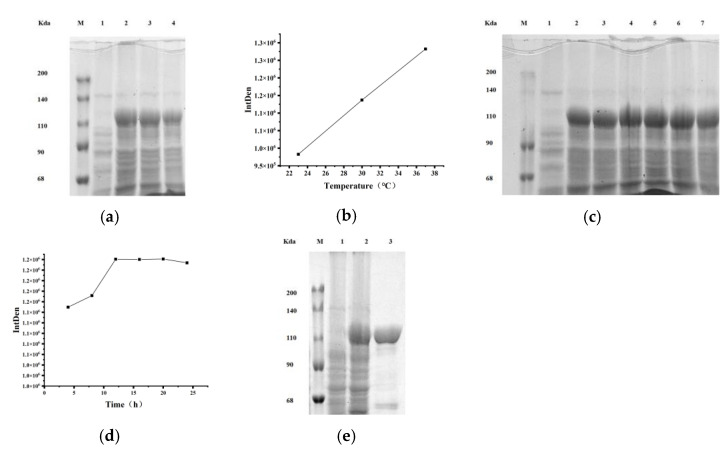
Electrophoretic analysis of induction temperature/time effects on enzyme expression and protein purification: (**a**) Induction Temperature Optimization. Lane M, protein marker; lane 1, uninduced pET28a-P450BM3, showing no target band; lane 2, induction at 37 °C, lane 3, induction at 30 °C, lane 4, induction at 20 °C. (**b**) Grayscale analysis of enzyme expression levels at different induction temperatures. (**c**) Induction duration optimization. Lane M, protein marker; lane 1, uninduced pET28a-P450BM3, lanes 2–7, induction for 4 h, 8 h, 12 h, 16 h, 20 h, and 24 h. (**d**) Grayscale analysis of enzyme expression levels under different Induction durations. (**e**) Protein purification verification. Lane M, protein marker; lane 1, uninduced pET28a-P450BM3; lane 2, induced pET28a-P450BM3, lane 3, purified cytochrome P450BM3.

SDS-polyacrylamide gel electrophoresis (SDS-PAGE) detected the target protein band of P450BM3 (≈119 kDa) in all induced groups of recombinant *E. coli* expressing P450BM3. When the induction time was set at 4 h, the highest protein yield occurred at 37 °C (Figure 2a,b). At an induction temperature of 37 °C, the maximum protein expression level was achieved after 12 h of induction (Figure 2c,d). SDS-PAGE also showed the successfully purified P450BM3 protein (≈119 kDa) (Figure 2e).

#### 2.1.3. Optimization of Cytochrome P450BM3 Catalytic Conditions for Gossypol Degradation

**Figure 3:**
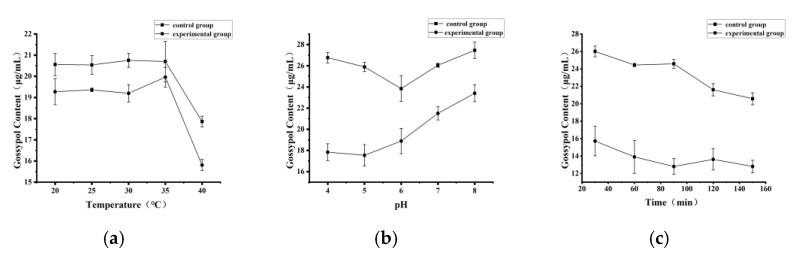
Optimization of reaction conditions for cytochrome P450BM3-catalyzed gossypol reduction: (**a**) Temperature optimization for cytochrome P450BM3-catalyzed gossypol reduction. (**b**) pH optimization for cytochrome P450BM3-catalyzed gossypol reduction. (**c**) Time optimization for cytochrome P450BM3-catalyzed gossypol reduction.

The enzyme activity of P450BM3 was found to be 1526.63 nmol/min/mg when palmitic acid served as the substrate. As depicted in Figure 3a–c, the optimal reaction conditions for the catalytic reaction of gossypol by P450BM3 were as follows: the reaction temperature was 30 °C, the pH value was 5.0, and the catalytic time was 90 min.

### 2.2. Site-Directed Mutagenesis of Cytochrome P450BM3 and Comparative Analysis of Gossypol Degradation

#### 2.2.1. Prediction of Catalytic Active Sites in Cytochrome P450BM3 for Gossypol Reduction

**Figure 4:**
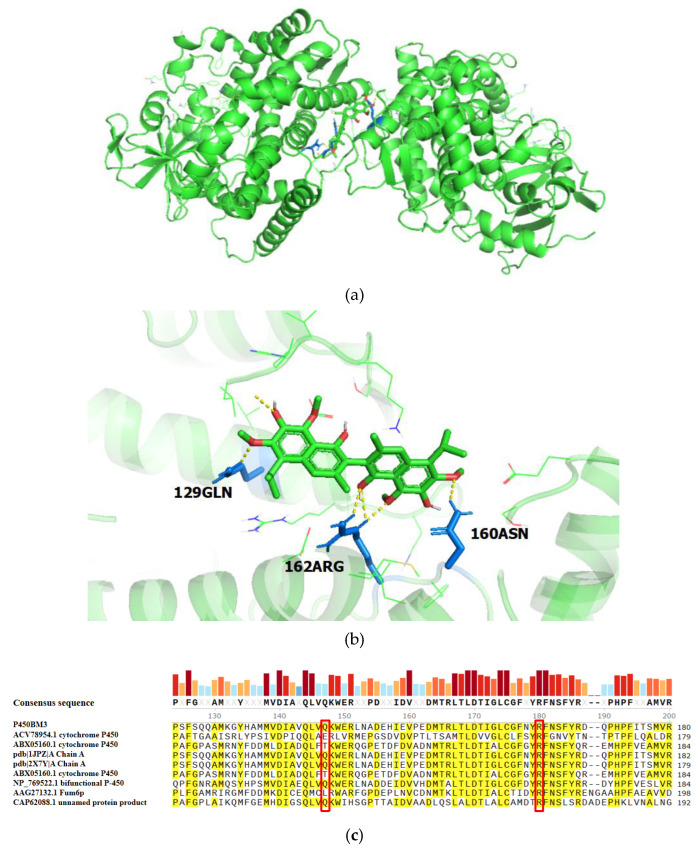
Prediction of catalytic active sites for gossypol in cytochrome P450BM3: (**a**) Cytochrome P450BM3. (**b**) Molecular docking model of gossypol and prediction of catalytic active sites in cytochrome P450BM3. (**c**) Conservation analysis of cytochrome P450BM3.

An activity-based analysis of molecular docking results (Table S1) showed that Site 1 had the highest binding affinity (−6.6 kcal/mol), the lowest inhibition constant (Ki = 14.53 μM), and the highest ligand efficiency (−0.16). The molecular docking model for Site 1 (Figure 4a) displayed an RMSD value of 1.6 Å relative to the reference structure. This 1.6 Å RMSD value was below the 2 Å threshold (Interpretation of RMSD values: RMSD < 2 Å indicates high consistency and reliable results; 2 Å < RMSD < 3 Å suggests moderate deviations but acceptable outcomes; RMSD > 3 Å reflects significant discrepancies and potentially unreliable results.), confirming substantial consistency between the docking and reference conformations. Predictive analysis (Figure 4b) screened out the key amino acid residues Q129, D160, and R162 that may be involved in the catalysis of gossypol. Conservation analysis (Figure 4c) results indicate that site 129 of cytochrome P450BM3 had a conservation score of 48, while site 162 had a conservation score of 100.

#### 2.2.2. Site-Directed Mutagenesis of Cytochrome P450BM3

**Figure 5:**
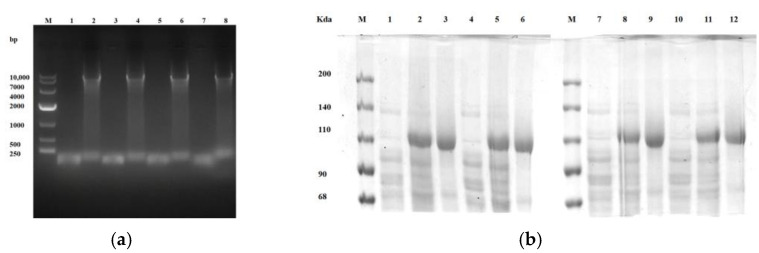
Electrophoretic analysis of P450BM3 site-directed mutagenesis PCR and induced expression/purification: (**a**) Lane M, DL10,000 DNA marker, lane 1, R162K PCR control, lane 2, R162K PCR product, lane 3, R162H PCR control, lane 4, R162H PCR product, lane 5, Q129N PCR control, lane 6, Q129N PCR product; lane 7, Q129H PCR control, lane 8, Q129H PCR product. (**b**) Lane M, protein marker, lane 1: uninduced R162K, lane 2, induced R162K, lane 3, purified R162K, lane 4, uninduced R162H; lane 5, induced R162H, lane 6, purified R162H, lane 7: uninduced Q129N, lane 8: induced Q129N, lane 9, purified Q129N, lane 10, uninduced Q129H; lane 11: induced Q129H; lane 12, purified Q129H.

Agarose gel electrophoresis of the PCR product showed that the target gene fragment was approximately 8 kb (Figure 5a). Sequencing verified successful site-directed mutagenesis. SDS-PAGE analysis of the induced expression and purification products presented expected results (Figure 5b).

#### 2.2.3. Comparison of Gossypol Degradation and Enzymatic Activity Between Cytochrome P450BM3 and Its Mutants

**Figure 6:**
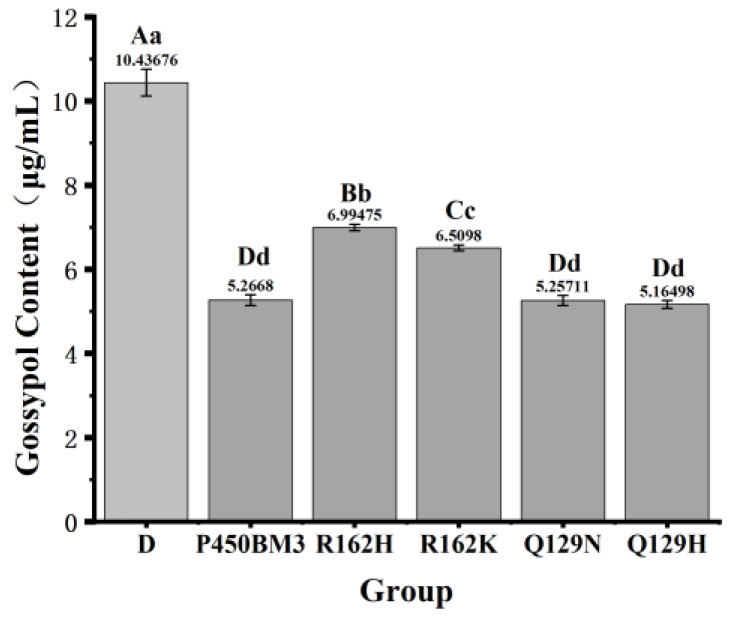
Comparison of gossypol detoxification and catalytic effect between cytochrome P450BM3 and its mutants. Note: Data within the same row labeled with no letters or identical letters indicate no significant difference (*p* > 0.05). Different lowercase letters denote significant differences (*p* < 0.05), while different uppercase letters indicate highly significant differences (*p* < 0.01). The same conventions apply hereafter. “D” denotes the control group; the same labeling applies hereafter.

As shown in Figure 6, P450BM3 and its mutants (R162H, R162K, Q129N, Q129H) significantly reduced gossypol levels compared with the control group (*p* < 0.01). Mutants Q129N and Q129H showed 0.1% and 1.2% increases in catalytic gossypol removal activity, respectively, relative to P450BM3. In contrast, mutants R162H and R162K exhibited 33.4% and 24.2% decreases in activity, respectively, compared with P450BM3.

### 2.3. Changes in Gossypol Metabolites Catalyzed by Cytochrome P450BM3 and Its Mutants

#### Screening of Differential Metabolites

Between the P450BM3-treated group and the control group, 56 differential metabolites were identified (Figure S6a,b). In positive ion mode, 13 metabolites were upregulated and 30 were downregulated in the P450BM3 group. In negative ion mode, 4 metabolites were upregulated and 22 were downregulated. Between the P450BM3 (R162H) mutant group and the control group, 57 differential metabolites were found (Figure S6c,d). In positive ion mode, 12 metabolites were upregulated and 33 were downregulated in the R162H mutant group. In negative ion mode, nine metabolites were upregulated and three were downregulated. For the P450BM3 (Q129H) mutant group versus the control group, 143 differential metabolites were identified (Figure S6e,f). In positive ion mode, 41 metabolites were upregulated and 76 were downregulated in the Q129H mutant group. In negative ion mode, 4 metabolites were upregulated and 22 were downregulated. Comparing the P450BM3 (R162H) mutant group with the wild-type P450BM3 group, 90 differential metabolites were detected (Figure S6g,h). In positive ion mode, 37 metabolites were upregulated and 36 were downregulated in the R162H mutant group. In negative ion mode, 11 metabolites were upregulated and 6 were downregulated. Between the P450BM3 (Q129H) mutant group and the wild-type P450BM3 group, 152 differential metabolites were found (Figure S6i,j). In positive ion mode, 47 metabolites were upregulated and 72 were downregulated in the Q129H mutant group. In negative ion mode, 7 metabolites were upregulated and 26 were downregulated.

**Figure 7:**
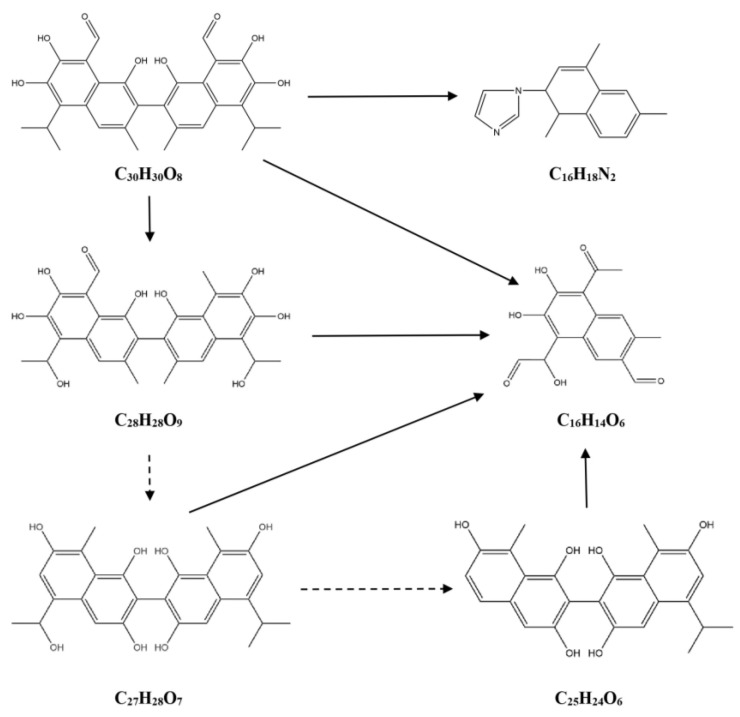
Degradation pathway of gossypol catalyzed by cytochrome P450BM3. Note: The dotted arrows indicate downregulation, whereas solid arrows denote upregulation of intermediate products; the same labeling applies hereafter.

**Figure 8:**
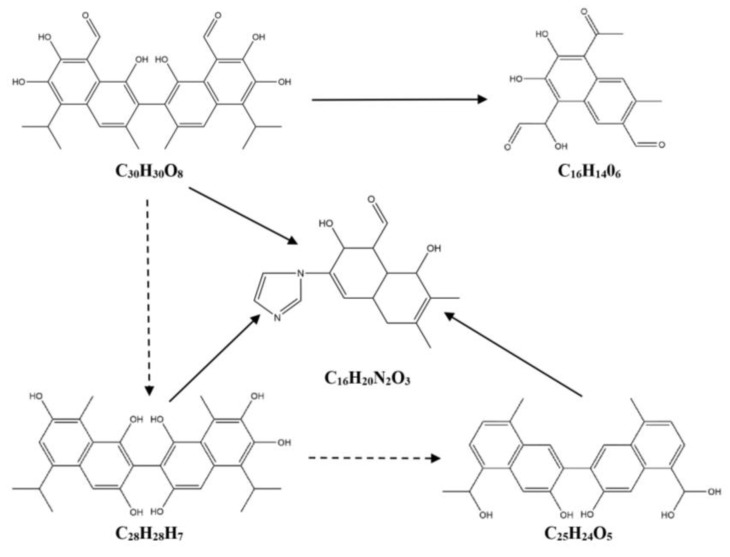
Degradation pathway of gossypol catalyzed by cytochrome P450BM3 (R162H).

**Figure 9:**
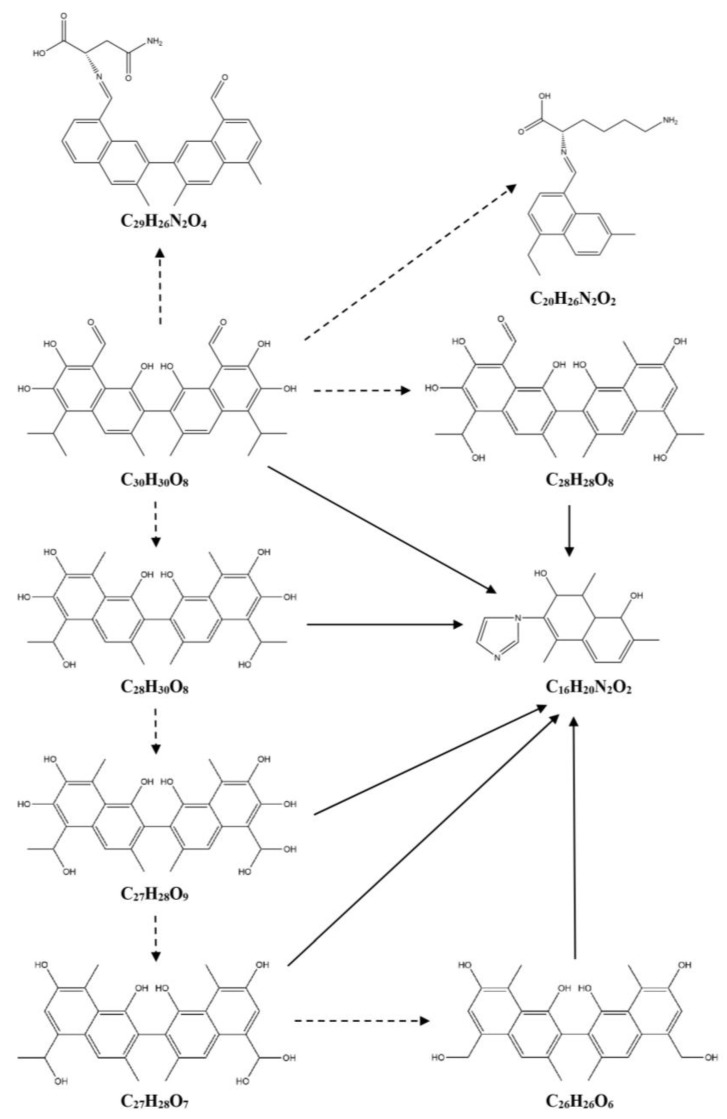
Degradation pathway of gossypol catalyzed by cytochrome P450BM3 (Q129H).

Based on the differential metabolites identified in this experiment, the degradation products of gossypol catalyzed by cytochrome P450BM3 and its mutants were hypothesized. As shown in Table 1 and Figure 7, there are three pathways for the degradation of gossypol catalyzed by the wild-type cytochrome P450BM3. In Pathway 1, C_30_H_30_O_8_ undergoes decarboxylation and hydroxylation to form C_28_H_28_O_9_. Then, C_28_H_28_O_9_, C_27_H_28_O_7_, and C_25_H_24_O_6_ further go through decarboxylation, dehydroxylation, and C-C cleavage to generate C_16_H_14_O_6_. In Pathway 2, C_30_H_30_O_8_ is converted into C_16_H_14_O_6_ through decarboxylation, hydroxylation, and C-C cleavage. In Pathway 3, C_30_H_30_O_8_ combines with an imidazole group after decarboxylation, dehydroxylation, and C-C cleavage to yield C_16_H_18_N_2_. As shown in Table 1 and Figure 8, there are two pathways for the degradation of gossypol catalyzed by the cytochrome P450BM3 (R162H) mutant. In Pathway 1, C_30_H_30_O_8_ is transformed into C_16_H_14_O_6_ via decarboxylation, hydroxylation, and C-C cleavage. In Pathway 2, C_30_H_30_O_8_, C_28_H_30_O_7_, and C_25_H_24_O_5_ combine with an imidazole group after decarboxylation, hydroxylation, and C-C cleavage to form C_16_H_20_N_2_O_3_. As shown in Table 1 and Figure 9, there are three pathways for the degradation of gossypol catalyzed by the cytochrome P450BM3 (Q129H) mutant. In Pathway 1, C_30_H_30_O_8_ combines with an amino acid after decarboxylation and dehydroxylation (along with C-C cleavage) to produce C_29_H_26_N_2_O_4_ and C_20_H_26_N_2_O_2_. In Pathway 2, C_30_H_30_O_8_, C_28_H_30_O_8_, C_28_H_28_O_8_, C_27_H_28_O_9_, C_27_H_28_O_7_, and C_26_H_26_O_6_ combine with an imidazole group after decarboxylation, hydroxylation, and C-C cleavage to generate C_16_H_20_N_2_O_2_.

## 3. Discussion

### 3.1. Construction and Expression of Cytochrome P450BM3 in E. coli

In this study, cytochrome P450BM3 was successfully expressed in *E. coli*, a strategy supported by previous investigations [22,23]. While low-temperature induction promotes proper folding of recombinant proteins, it restricts bacterial growth and overall enzyme yield. Conversely, higher induction temperatures enhance cellular growth but carry the risk of inclusion body formation and associated loss of catalytic activity, primarily due to the lack of eukaryotic folding chaperones in *E. coli* [24]. This deficiency leads to the aggregation of hydrophobic domains in P450BM3, thereby impairing its catalytic activity. Prior studies have indicated that 30 °C represents the optimal induction temperature for P450BM3 activity in *E. coli*, a parameter aligned with the native host *Bacillus megaterium*’s optimal growth temperature [25]. Thus, 30 °C was selected as the induction temperature, consistent with findings in studies of CYP102A16 [26]. Induction duration was identified as a critical factor for expression: prolonged induction disrupts cellular metabolism, leading to cell lysis or protein degradation [27]. A 12 h induction period was determined to be optimal, consistent with prior research [26]. The purified P450BM3 displayed a ~119 kDa band on SDS-PAGE, in line with earlier findings [28]. Therefore, appropriate induction temperature and induction time are more conducive to the expression of the protein.

### 3.2. Optimization of Cytochrome P450BM3-Catalyzed Gossypol Degradation Conditions

P450BM3 is a versatile enzyme. Its catalytic activity was first verified using palmitic acid as a model substrate before being applied to gossypol reduction, a validation approach consistent with previous research [13,29]. Since enzymatic efficiency depends on multiple factors, temperature, pH, and reaction time were optimized systematically. The optimal conditions for gossypol degradation were found to be 30 °C, pH 5.0, and 90 min. For instance, a prior study reported that the optimal conditions for *Helicoverpa armigera* CYP9A12 were 30 °C and pH 6.0 [12]. Such differences in optimal pH values likely result from variations in substrate–enzyme specificity among P450 isoforms. In the context of other cytochrome P450-related studies, various reaction conditions and efficiencies have been documented. At pH 7 and 35 °C, P450BM3 achieved a 44.08% indigo conversion [25]. CYP119 showed a kcat of 78.2 min^−1^ for styrene oxidation at 70 °C and pH 8.5 [30]. CYP102A16 degraded 34.9% of 50 ppm naphthalene at 37 °C within 60 h, while P450DA-G4 demonstrated 76% hydroxylation efficiency for 1-chloro-2-phenylethane at 30 °C and pH 8.5 [26,31]. These diverse findings underscore the significant influence of substrate chemistry and enzyme–substrate interactions on reaction conditions. By using purified P450BM3, potential interference from amines and proteins in crude lysates was eliminated, confirming the enzyme’s direct role in reducing gossypol levels.

### 3.3. Comparison of Catalytic Efficiency Between Cytochrome P450BM3 and Its Mutants

Molecular docking models were employed to predict key residues within P450BM3’s active site, enabling the generation of mutants P450BM3 (R162H), (R162K), (Q129N), and (Q129H) via site-directed mutagenesis. While Q129N and Q129H exhibited no significant alterations in gossypol reduction efficiency compared with the wild-type enzyme, R162H and R162K displayed 33.4% and 24.2% reductions in catalytic efficiency, respectively. These results underscore the critical role of Arg129 in catalytic function. Analogous trends have been reported in other enzymatic systems. For instance, the Thermobifida fusca cutinase mutant H129A showed diminished PET hydrolysis efficiency [32]. In Ideonella sakaiensis PETase, the S178T and S209V mutants retained only 29.7% and 38.2% of the wild-type efficiency, respectively [33]. Additionally, the ROC GTP (N1437H) mutant demonstrated a two-fold decrease in GTP hydrolysis efficiency [34]. Based on these findings, the R162H and Q129H mutants were selected for further metabolomic analysis of gossypol reduction, aiming to unravel the underlying catalytic mechanisms.

### 3.4. Degradation Products and Pathways of Gossypol Catalyzed by Cytochrome P450BM3 and Its Mutants

Current research has demonstrated that P450BM3, a highly versatile enzyme, is capable of significantly reducing gossypol levels in reaction systems. As such, further investigation into gossypol degradation pathways and structural elucidation of its degradation products are imperative. Gossypol derivatives can be synthesized through reactions such as Schiff base formation [35], ozonation [36], oxidation [37], and methylation [11]. However, the current understanding of enzymatic gossypol degradation remains limited, with proposed pathways exhibiting substantial variability.

Laccase has been shown to catalyze the intramolecular cyclization of aldehyde and hydroxyl groups in gossypol, leading to the formation of o-semiquinone radicals and the release of hydroxyl (OH) radicals [11]. In *Helicoverpa armigera*, gossypol undergoes CYP9A12-mediated oxidative demethylation and hydroxylation, generating intermediate metabolites G1 (*m*/*z* 265.14, C_12_H_18_O_7_) and G2 (*m*/*z* 293.17, C_15_H_18_O_6_), which are further oxidized to the final products G0 (*m*/*z* 209.08, C_12_H_18_O_3_) and G0′ (*m*/*z* 248.95, C_14_H_18_O_4_) [12]. Four metabolic pathways for gossypol degradation by Helicoverpa carboxylesterases have been described: hydrolysis of ester/amide bonds to yield carboxylic acids and alcohols; aldehyde–amine reactions forming hydrazones or Schiff bases (*m*/*z* 600.25, C_34_H_36_N_2_O_6_); substitution/elimination of unstable hydroxyl groups to produce less toxic products (*m*/*z* 488.26, C_31_H_36_O_5_); and covalent binding with amines to form macromolecular complexes (*m*/*z* 713.46, C_47_H_59_N_3_O_3_) [13]. Additionally, *H. armigera* UGT41B3 and UGT40D1 have been identified to catalyze glycosylation by transferring glycosyl groups from UDP-glucose to gossypol’s hydroxyl or aldehyde moieties, resulting in diglycosylated gossypol isomer 5 [14]. Collectively, these findings highlight the enzymatic diversity underlying gossypol detoxification and underscore the complexity of reduction pathways derived from microbial and insect sources. Proposed mechanisms—including oxidative modifications (hydroxylation, demethylation) and covalent conjugation (Schiff base formation, glycosylation)—provide a foundational framework for understanding gossypol degradation processes across diverse enzymatic systems.

Differentially abundant metabolites distinct from the control group were identified, and reduction products and pathways were proposed based on metabolomic data. Metabolomic evidence indicates that gossypol generates reduction products—including C_28_H_28_O_9_, C_27_H_28_O_7_, C_25_H_24_O_6_, C_16_H_14_O_6_, C_16_H_18_N_2_, C_28_H_30_O_7_, C_25_H_24_O_5_, C_16_H_20_N_2_O_3_, C_28_H_30_O_8_, C_28_H_28_O_8_, C_27_H_28_O_9_, C_27_H_28_O_7_, C_26_H_26_O_6_, C_20_H_26_N_2_O_2_, C_29_H_26_N_2_O_4_, and C_16_H_20_N_2_O_2_—primarily through decarboxylation, hydroxylation, and C-C bond cleavage pathways under the catalysis of P450BM3 and its mutants. Notably, the reduction products C_28_H_28_O_9_, C_28_H_30_O_8_, and C_28_H_28_O_8_ align with intermediates proposed in studies of gossypol degradation by *Candida parapsilosis* KDN0118 [38]. Unlike previous studies, this study analyzed the trends in changes in intermediate products. Under the action of the enzyme, the production of C_28_H_28_O_9_ was upregulated, while the production of C_28_H_30_O_8_ and C_28_H_28_O_8_ was downregulated. Additionally, four detoxification products (C_28_H_30_O_6_, C_30_H_26_O_10_, C_30_H_26_O_12_, and C_12_H_10_O_7_) were isolated from liver tissues using ^14^C-labeled gossypol, with two metabolic pathways previously proposed [39]. A hypothesized pathway involving decarboxylation of C_30_H_30_O_8_ to C_28_H_30_O_6_ is consistent with decarboxylation mechanisms proposed here for P450BM3 and its mutants. Moreover, decarboxylation of C_30_H_30_O_8_ to yield C_28_H_30_O_6_, along with the generation of C_15_H_18_O_6_ through decarboxylation, hydroxylation, and C-C cleavage, corroborates pathways associated with the mutants Q129H and R162H [12]. Previous studies have hypothesized that the aldehyde group in gossypol reacts with the amino group of amino acids in *Helicoverpa armigera* carboxylesterase to form hydrazones or Schiff bases [13]. Additionally, research on the interaction between amino acids and gossypol has shown that gossypol can generate binding products with amino acids [40,41,42], which is consistent with the pathway proposed in this study for the binding of amino acids in the enzyme to gossypol. Gossypol can bind to the imidazole group of histidine [43], which is consistent with the hypothesized pathway in this study, where gossypol degradation intermediates bind to imidazole. Additionally, this study also found that the putative intermediates C_16_H_18_N_2_ and C_16_H_20_N_2_O_2_ in gossypol reduction share structural similarities with the standard compound 1-[(2-methyl-1H-imidazol-1-yl)methyl]-2-naphthol. The current mechanistic understanding of enzymatic gossypol degradation remains incomplete. The inferred gossypol reduction metabolites and removal pathways in this study may only represent part of those mediated by cytochrome P450BM3 and its mutants. The hypothesized reduction metabolites and pathways currently require further in-depth validation and analysis, and whether the gossypol reduction metabolites are toxic remains to be verified. Additionally, the reliance of cytochrome P450BM3 on exogenous NADPH supplementation to sustain catalytic activity increases operational costs, necessitating the exploration of alternative enzymes or cost-mitigation strategies for practical industrial-scale applications.

## 4. Conclusions

This study found that cytochrome P450BM3 and its four mutants—P450BM3 (R162H), P450BM3 (R162K), P450BM3 (Q129N), and P450BM3 (Q129H)—significantly reduced gossypol levels. Compared with the wild-type enzyme, the catalytic efficiencies of R162H and R162K were reduced by 33.4% and 24.2%, respectively. Therefore, it is speculated that arginine 162 (Arg162) is highly likely to be a key active residue in the catalytic reduction of gossypol. Based on metabolomic findings, it is hypothesized that cytochrome P450BM3 and its mutants (R162H, Q129H) catalyze the formation of gossypol reduction intermediates primarily through reaction pathways involving decarboxylation, hydroxylation, and C-C bond cleavage. The enzyme contributes to reducing gossypol levels, but its precise catalytic mechanism remains to be further investigated.

## 5. Materials and Methods

### 5.1. General

Components for culture media were purchased from Qingdao Haibo Biotechnology Co, Ltd. (Qingdao, China). Restriction enzymes, PCR kits, and cloning reagents were purchased from Jinsha Biotechnology (Shanghai, China) and Takara Bio (Shiga, Japan). NADPH-Na4 was purchased from Sangon Biotech (Shanghai, China). Gossypol was purchased from APExBIO (Houston, TX, USA). All chemicals were of analytical grade or higher quality.

### 5.2. Bacterial Strains, Plasmids, and DNA Techniques

*B. megaterium* ATCC 14581 and expression vector pET28a were maintained by the Xinjiang Key Laboratory of Herbivore Nutrition for Meat & Milk, Xinjiang Agricultural University (Urumqi, China). *E. coli* DH5α (Sangon Biotech, Shanghai, China) was used for plasmid propagation. *E. coli* BL21 (DE3) (Tiangen Biotech, Beijing, China) functioned as the heterologous expression host. Genomic DNA of *B. megaterium* ATCC 14581 was extracted using the Bacterial DNA Extraction Kit (Tiangen Biotech, Beijing, China) after overnight culture in LB medium at 37 °C (150 rpm). Primers P1 (5′-AAAACGACGGCCAGTATGACAATTAAAGAAATGCCTCAGC-3′) and P2 (5′-GGAAACAGCTATGACTTACCCAGCCCACACGTC-3′) were designed based on the *P450BM3* gene sequence (GenBank accession No. J04832.1). The cloning vector pMD19-T (Takara Bio, Shiga, Japan) was linearized by PCR using primers P3 (5′-ACTGGCCGTCGTTTTAC-3′) and P4 (5′-GTCATAGCTGTTTCCTG-3′). The P450BM3 gene was amplified from *B. megaterium* genomic DNA using 1.1× S4 Fidelity PCR Mix (Jinsha Biotechnology, Shanghai, China). PCR products were gel-purified and seamlessly cloned into pMD19-T using Solarbio In-Fusion^®^ Snap Assembly Master Mix (Takara Bio, Shiga, Japan). Recombinant plasmids (pMD19T-P450BM3) were transformed into *E. coli* DH5α and validated by blue-white screening, PCR, and sequencing. The pET28a plasmid was linearized by double digestion with *BamH*I and *EcoR*I. The P450BM3 gene was amplified from pMD19T-P450BM3 using primers P5 (5′-AGCAAATGGGTCGCGGATCCATGACAATTAAAGAAATGCCTCAGCC-3′) and P6 (5′-TGTCGACGGAGCTCGAATTCTTACCCAGCCCACACGTC-3′), introducing *BamH*I and *EcoR*I restriction sites, the PCR product was gel-purified and assembled with linearized pET28a via seamless cloning [44]. Recombinant pET28a-P450BM3 was transformed into *E. coli* BL21 (DE3). Positive clones were confirmed by restriction digestion and sequencing.

### 5.3. Protein Expression and Purification

The seed culture was inoculated into LB liquid medium at 1% (*v*/*v*) and cultivated in a shaking incubator at 37 °C and 220 rpm until the optical density (OD_600_) reached approximately 0.8, after which gene expression was induced by adding 0.5 mM isopropyl-β-D-thiogalactopyranoside (IPTG) [45]. Temperature optimization involved induction at 23 °C, 30 °C, and 37 °C for 8 h, while duration optimization tested induction times of 4, 8, 12, 16, 20, and 24 h under the optimal temperature determined from the temperature optimization experiment. Expression profiles were quantitatively analyzed using ImageJ software 1.8.0 (NIH, Bethesda, MD, USA). Post-induction cells were harvested by centrifugation at 8000× *g* for 10 min at 4 °C (Sigma 3K15, Sigma Laborzentrifugen GmbH, Osterode, Germany), washed with phosphate-buffered saline (PBS), and resuspended in PBS. Crude enzyme extract was obtained after a secondary centrifugation step at 8000× *g* for 25 min at 4 °C (Sigma 3K15, Sigma Laborzentrifugen GmbH, Osterode, Germany). The extract was purified via Ni-NTA (Sangon Biotech, Shanghai, China) affinity chromatography (Binding/Wash Buffer: 20 mM imidazole buffer; Elution Buffer: 250 mM imidazole buffer) using a Ni-NTA column, followed by desalting through a Sephadex Gravity Desalting Column from the same supplier. Protein concentration was determined using a Micro BCA Protein Assay Kit (Sangon Biotech, Shanghai, China). SDS-PAGE analysis was conducted using 6% polyacrylamide gels.

### 5.4. Cytochrome P450BM3 Enzyme Activity Measurements

The activity of P450BM3 enzymes was determined by the NADPH oxidation assay [28]. The 1 mL reaction mixture contained 0.25 mM palmitic acid in DMSO (final concentration, 2%) and 50 μg P450BM3 enzyme in 100 mM PBS (pH 7.4). The reaction was started by adding 15 μL 10 mM aqueous NADPH-Na4 solution, followed by 340 nm (eNADPH = 6.22 mM^−1^ cm^−1^). Enzyme activity unit: One unit (U) of enzyme activity was defined as the amount of P450BM3 required to consume 1 nmol of NADPH per minute at 25 °C.

### 5.5. Examination of the Catalytic Effect of Cytochrome P450BM3 on Gossypol

To investigate the effect of the recombinant P450BM3 enzymes on gossypol reduction, the reactions were performed in 1 mL of 100 mM PBS (pH 7.0) with 50 μL of gossypol (30 μg) and 50 μg of the enzyme protein. The reaction was initiated by the addition of 15 μL 10 mM aqueous NADPH-Na4 solution, which is the co-factor for P450 monooxygenase [46]. The reaction mixtures were incubated for 30 min at 30 °C. The prepared samples were quantified by the extraction of 70% aqueous acetone on a shaker for 1 h and then centrifuged for 1 min at 4 °C, 12,000× *g* (SCILOGEX CF1524R, SCILOGEX, Rocky Hill, CT, USA). The supernatant was collected and analyzed by HPLC.

With an aim to optimize, the following actions were executed: for pH optimization, reaction mixtures at pH 4.0, 5.0, 6.0, 7.0, and 8.0 were incubated in a 30 °C water bath for 30 min before measuring the residual gossypol content post P450BM3 catalysis; for temperature optimization, reaction mixtures with pH 7.0 were incubated at 20 °C, 25 °C, 30 °C, 35 °C, and 40 °C for 30 min and then the residual gossypol content was quantified; and for time optimization, reaction mixtures at pH 5.0 and 30 °C were incubated for 30, 60, 90, 120, and 150 min, and the residual gossypol content was measured at each time point.

### 5.6. HPLC Determination of Gossypol

HPLC (Shimadzu LC-40, Shimadzu Corporation, Kyoto, Japan) conditions were adapted from Rahma et al. [47]. Analytical column: C18 column (250 mm × 4.6 mm, 5 μm). Mobile phase: Acetonitrile: 0.2% phosphoric acid (85:15, *v*/*v*). Flow rate: 1 mL/min. Detection wavelength: 235 nm. Injection volume: 20 μL. Column temperature: 30 °C.

### 5.7. Site-Directed Mutagenesis of Cytochrome P450BM3

Molecular docking between cytochrome P450BM3 (PDB ID: 6K3Q) and gossypol (PubChem CID: 3503) was performed using AutoDock Vina 1.2.5 (The Scripps Research Institute, La Jolla, CA, USA). The docking models were visualized and analyzed with PyMOL. Meanwhile, conservation analysis of cytochrome P450BM3 was carried out using SnapGene 6.0.2 (GSL Biotech, Boston, MA, USA). Based on the results of molecular docking and conservation analysis, site-directed mutagenesis was conducted using a one-step PCR method [48]. Glutamine at position 129 (Q129) of P450BM3 was randomly mutated to histidine (H) or asparagine (N), and arginine at position 162 (R162) was mutated to histidine (H) or lysine (K). Primers R162KP1 (5′-TGTTAAATTTATAGTTAAAGCCGCAAAGACC-3′), R162KP2 (5′-ACTATAAATTTAACAGCTTTTACCGAGATCAGC-3′); R162HP1 (5′-TGTTAAAGTGATAGTTAAAGCCGCAAAGACC-3′), R162HP2 (5′-ACTATCACTTTAACAGCTTTTACCGAGATCAGC-3′); Q129HP1 (5′-CCCACTTATGAACAAGCTGCACGGCGATATC-3′), Q129HP2 (5′-TTGTTCATAAGTGGGAGCGTCTAAATGCAG-3′); Q129NP1 (5′-CCCACTTGTTAACAAGCTGCACGGCGATATC-3′), Q129NP2 (5′-TTGTTAACAAGTGGGAGCGTCTAAATGCAG-3′) were designed using plasmid pET28(+)-P450BM3 as the template. PCR-amplified products were directly treated with the restriction enzyme *Dpn*I. The digested products were transformed into *E. coli* BL21(DE3) competent cells. Single colonies grown on antibiotic-containing LB agar plates were picked, cultured in LB liquid medium, and subjected to plasmid extraction and sequencing. Mutant strains were validated by sequencing.

### 5.8. Comparative Analysis of Catalytic Effect on Gossypol and Metabolite Detection by Cytochrome P450BM3 Enzyme and Its Mutants

Residual gossypol content was measured after the reaction between cytochrome P450BM3 enzyme and its mutants and gossypol. Subsequently, the samples were analyzed via ultra-performance liquid chromatography coupled with quadrupole time-of-flight mass spectrometry (UPLC-Q-TOF/MS) (Vanquish UHPLC/Q Exactive™ HF, Thermo Fisher, Dreieich, Germany) at Novogene Co., Ltd. (Beijing, China).

### 5.9. Data Analysis

One-way ANOVA was performed using SPSS 19.0 (SPSS Inc., Chicago, IL, USA), and Duncan’s method was used for significant difference analysis (*p* < 0.05). All data were expressed as mean ± SD. The raw metabolomics data files were converted to mzXML format using ProteoWizard. Metabolite identification was performed via XCMS [49] based on full-scan analysis and extracted ion chromatogram (XIC) analysis of mass spectrometry data. Data processing was conducted on a Linux operating system (CentOS version 6.6) using R 4.2.3 and Python 3.1.0, and chemical structural formulas were drawn with ChemDraw 22.0 software (CambridgeSoft, Cambridge, MA, USA).

## Figures and Tables

**Table 1:** Proposed degradation products of gossypol catalyzed by cytochrome P450BM3.

Group	Item	Compound	*m*/*z*	RT (min)	Regulate
P450BM3	1	C_28_H_28_O_9_	507.16	6.64	Up
	2	C_27_H_28_0_7_	465.19	6.79	Down
	3	C_25_H_24_O_6_	419.15	6.76	Down
	4	C_16_H_14_O_6_	301.07	6.98	Up
	5	C_16_H_18_N_2_	239.15	5.50	Up
R162H	1	C_28_H_30_O_7_	477.19	6.96	Down
	2	C_25_H_24_O_5_	405.17	6.76	Down
	3	C_16_H_14_O_6_	301.07	6.98	Up
	4	C_16_H_20_N_2_O_3_	367.18	6.82	Up
Q129H	1	C_28_H_30_O_8_	493.19	6.45	Down
	2	C_28_H_28_O_8_	493.19	6.76	Down
	3	C_27_H_28_O_9_	497.18	6.25	Down
	4	C_27_H_28_O_7_	465.20	6.12	Down
	5	C_26_H_26_O_6_	435.18	6.67	Down
	6	C_20_H_26_N_2_O_2_	349.18	9.33	Down
	7	C_29_H_26_N_2_O_4_	457.20	6.54	Down
	8	C_16_H_20_N_2_O_2_	273.16	5.57	Up

## Data Availability

The original contributions presented in this study are included in the article/Supplementary Materials. Further inquiries can be directed to the corresponding author.

## Supplementary Materials

The following supporting information can be downloaded at https://www.mdpi.com/article/10.3390/toxins17050253/s1, Figure S1: PLS-DA score scatter plots and permutation validation plots for metabolites in the cytochrome P450BM3-treated group and control group (D group); Figure S2: PLS-DA score scatter plots and permutation validation plots for metabolites in the R162H group and D group; Figure S3: PLS-DA score scatter plots and permutation validation plots for metabolites in the cytochrome P450BM3 (Q129H) group and D group; Figure S4: PLS-DA score scatter plots and permutation validation plots for metabolites in the P450BM3 group and R162H group; Figure S5: PLS-DA score scatter plots and permutation validation plots for metabolites in the P450BM3 group and R162H group; Figure S6: Volcano plots of differential metabolites; Table S1: Molecular docking model binding site prediction score.

## Abbreviations

The following abbreviations are used in this manuscript:

FG	Free gossypol
BG	Bound gossypol
CYP	Cytochrome P450
UPLC-Q-TOF/MS	Ultra-performance liquid chromatography–quadrupole time-of-flight mass spectrometry
