# Supplementary material for: Heterologous Overexpression of Cytochrome P450BM3 from Bacillus megaterium and Its Role in Gossypol Reduction
Source: Toxins (Basel). 2025 May 20;17(5):253. doi: 10.3390/toxins17050253 (PMC12115826; doi:10.3390/toxins17050253)
Supplement: Supplementary file 1 [file toxins-17-00253-s001.zip › toxins-3622369-supplementary.pdf]

1 Prediction of Catalytic Active Sites in Cytochrome P450BM3 for Gossypol Degradation

Table S1 Molecular docking model binding site prediction score

Binding Site	Affinity (kcal/mol)	Estimated Ki (uM)	Ligand Efficiency
1	-6.6	14.53	-0.16
2	-6.4	20.36	-0.16
3	-6.2	28.53	-0.15
4	-6.2	28.53	-0.15
5	-6.1	33.78	-0.15
6	-6.1	33.78	-0.15
7	-5.9	47.35	-0.15
8	-5.9	47.35	-0.15
9	-5.8	56.05	-0.14

Note : Binding site : represents different potential binding sites or docking conformation number. Affinity : The unit is kcal / mol, and the negative value indicates that the binding is a spontaneous process. The greater the absolute value, the stronger the binding. Estimated Ki (inhibition constant) : represents the dissociation constant of ligand binding to the receptor. The smaller the value, the higher the affinity. Ligand Efficiency : Measure the contribution of the unit atom of the ligand to the binding energy. The larger the absolute value of the negative value, the higher the efficiency.

2 PLS-DA of Cytochrome P450BM3-Catalyzed Gossypol Degradation

PLS-DA identified significant metabolomic differences between groups. Score scatter plots (Figures S1a, S1b) showed clear group separation under both ionization modes. Model parameters in positive mode were $R^2Y = 0.97$ and $Q^2Y = 0.87$; in negative mode, $R^2Y = 0.82$ and $Q^2Y = -0.79$, indicating robust group differentiation. Permutation validation (Figures S1c, S1d) confirmed model reliability, with $R^2 = 0.97/Q^2 = 0.87$ (positive) and $R^2 = 0.99/Q^2 = -0.84$ (negative), demonstrating strong predictive capacity.

3 PLS-DA of Cytochrome P450BM3 (R162H)-Catalyzed Gossypol Degradation

PLS-DA identified significant metabolomic differences between groups. Score plots (Figures S2a, S2b) showed clear separation under both modes. Model parameters were $R^2Y = 0.99/Q^2Y = 0.70$ (positive) and $R^2Y = 0.98/Q^2Y = 0.78$ (negative), confirming phenotype differences. Permutation validation (Figures S2c, S2d) supported reliability, with $R^2 = 0.78/Q^2 = -1.08$ (positive) and $R^2 = 0.92/Q^2 = -0.78$ (negative).

4 PLS-DA of Cytochrome P450BM3 (Q129H)-Catalyzed Gossypol Degradation

PLS-DA identified significant metabolomic differences between groups. Score plots (Figures S3a, S3b) showed group separation in both modes. Model parameters were $R^2Y = 1.00/Q^2Y = 0.96$ (positive) and $R^2Y = 0.99/Q^2Y = 0.96$ (negative), confirming phenotype differences. Permutation validation (Figures S3c, S3d) confirmed reliability, with $R^2 = 0.86/Q^2 = -0.75$ (positive) and $R^2 = 0.72/Q^2 = -1.32$ (negative).

5 Comparative PLS-DA of Gossypol Degradation Catalyzed by Cytochrome P450BM3 and P450BM3 (R162H)

PLS-DA identified significant metabolomic differences between groups. Score plots (Figures S4a, S4b) showed group separation in both modes. Model parameters were $R^2Y = 0.99/Q^2Y = 0.86$ (positive) and $R^2Y = 0.99/Q^2Y = 0.90$ (negative). Permutation validation (Figures S4c, S4d) confirmed reliability, with $R^2 = 0.86/Q^2 = -0.90$ (positive) and $R^2 = 0.71/Q^2 = -1.01$ (negative).

6 Comparative PLS-DA of Gossypol Degradation Catalyzed by Cytochrome P450BM3 and P450BM3 (Q129H)

PLS-DA identified significant metabolomic differences between groups. Score plots (Figures S5a, S5b) showed group separation in both modes. Model parameters were $R^2Y = 1.00/Q^2Y = 0.95$ (positive) and $R^2Y = 0.99/Q^2Y = 0.95$ (negative). Permutation validation (Figures S5c, S5d) confirmed reliability, with $R^2 = 0.81/Q^2 = -1.00$ (positive) and $R^2 = 0.61/Q^2 = -1.14$ (negative).

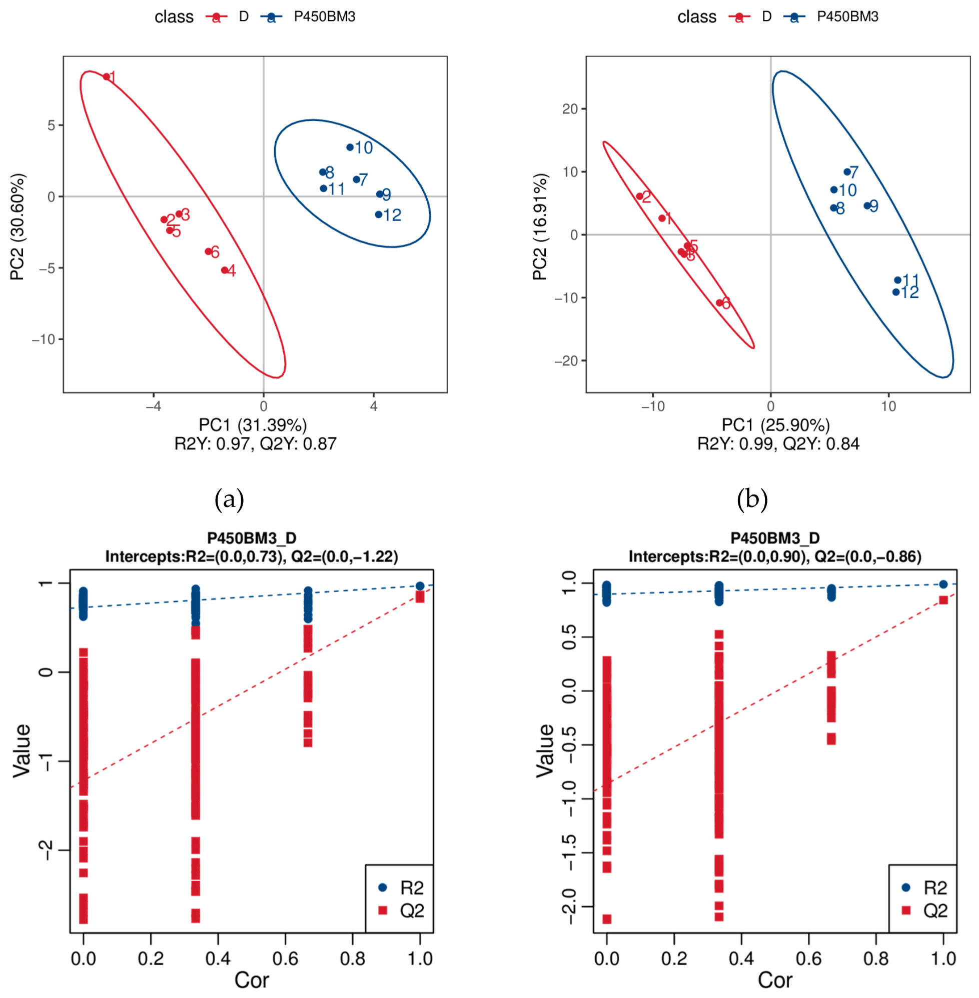

(c) (d)

Figure S1. PLS-DA score scatter plots and permutation validation plots for metabolites in the cytochrome P450BM3-treated group and control group (D group): (a) Positive ion mode score scatter plot. (b) Negative ion mode score scatter plot. (c) Positive ion mode permutation validation plot. (d) Negative ion mode permutation validation plot. "D" denotes the control group; the same labeling applies hereafter.

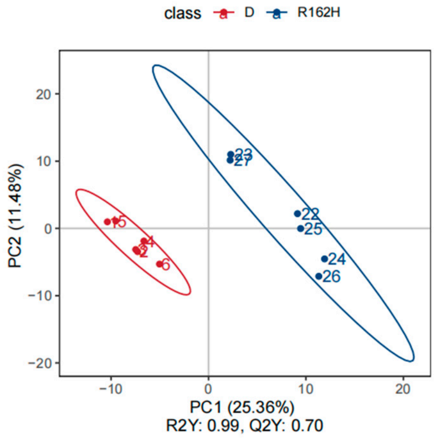

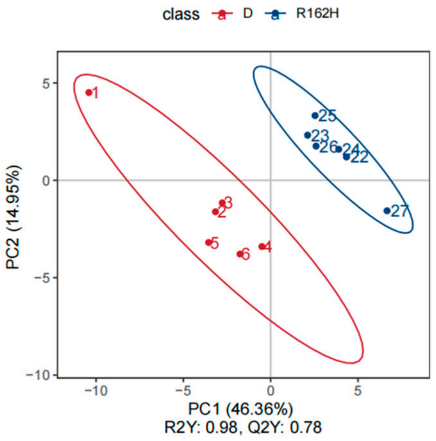

(a) (b)

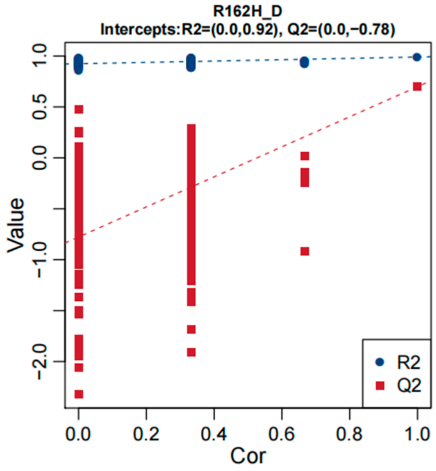

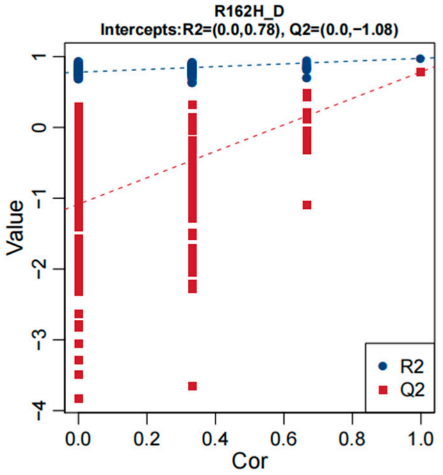

(c) (d)

Figure S2. PLS-DA score scatter plots and permutation validation plots for metabolites in the R162H group and D group: (a) Positive ion mode score scatter plot. (b) Negative ion mode score scatter plot. (c) Positive ion mode permutation validation plot. (d) Negative ion mode permutation validation plot.

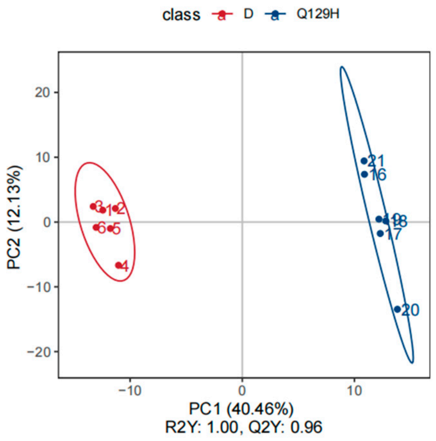

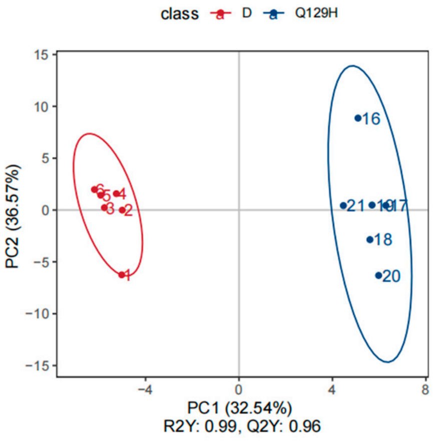

(a) (b)

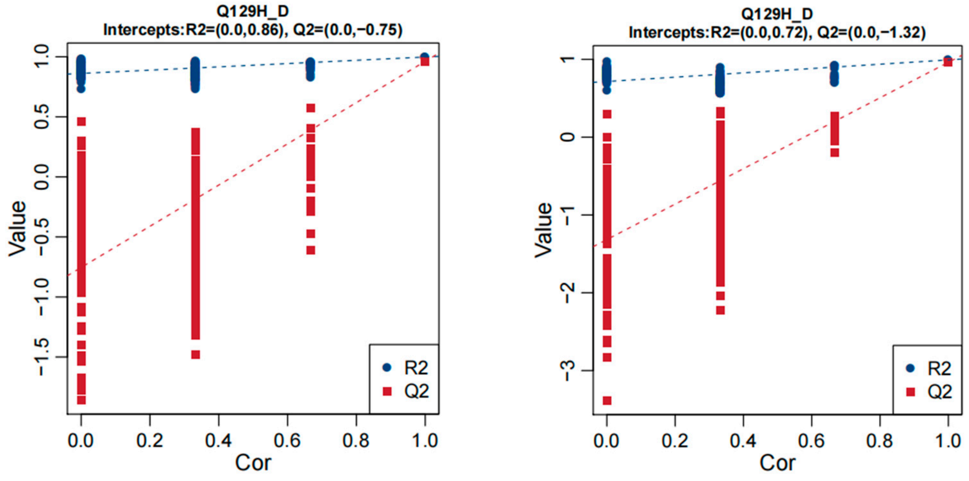

(c) (d)

Figure S3. PLS-DA score scatter plots and permutation validation plots for metabolites in the cytochrome P450BM3 (Q129H) group and D group: (a) Positive ion mode score scatter plot. (b) Negative ion mode score scatter plot. (c) Positive ion mode permutation validation plot. (d) Negative ion mode permutation validation plot.

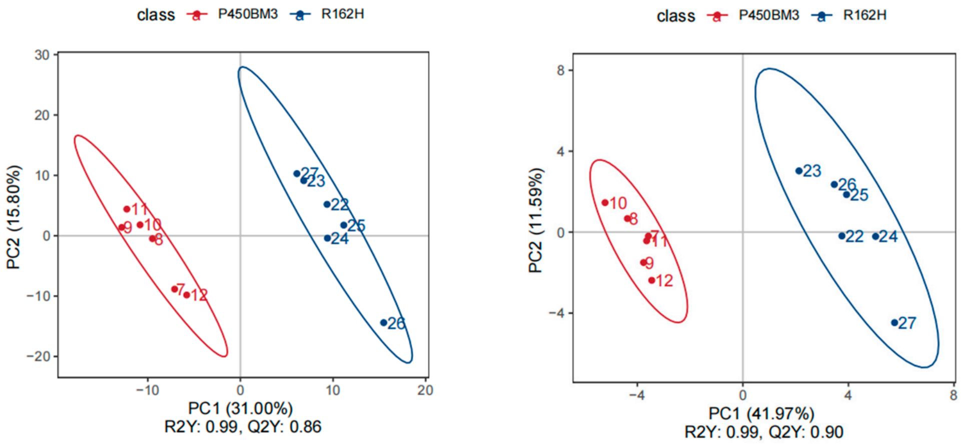

(a) (b)

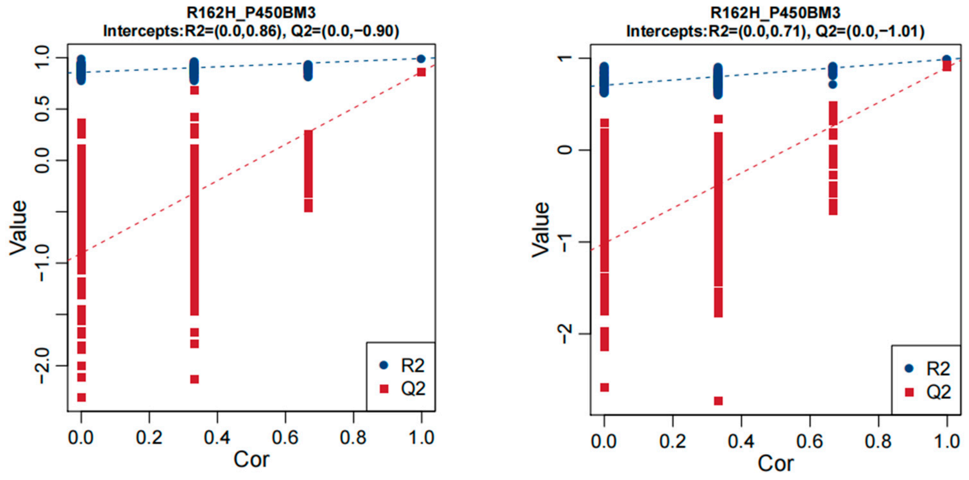

(c) (d)

Figure S4. PLS-DA score scatter plots and permutation validation plots for metabolites in the P450BM3 group and R162H group: (a) Positive ion mode score scatter plot. (b) Negative ion mode score scatter plot. (c) Positive ion mode permutation validation plot. (d) Negative ion mode permutation validation plot.

12 Partial least squares discriminant analysis (PLS-DA) of gossypol degradation catalyzed by cytochrome P450BM3 and P450BM3 (Q129H)

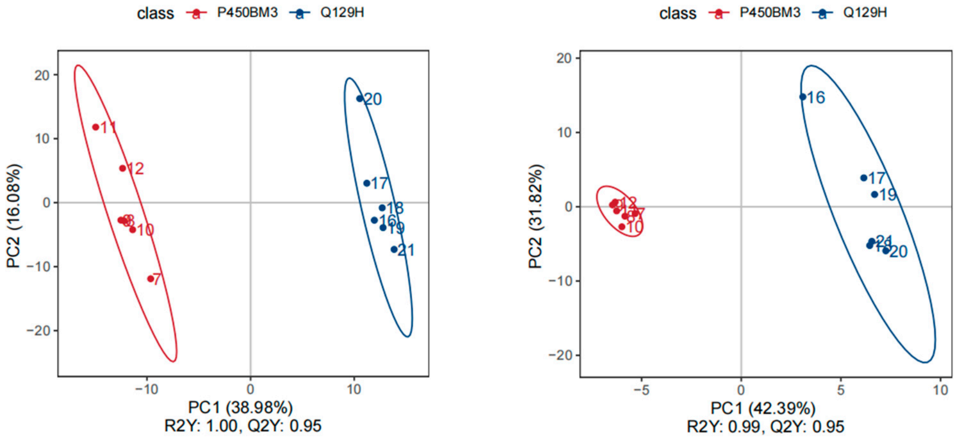

(a) (b)

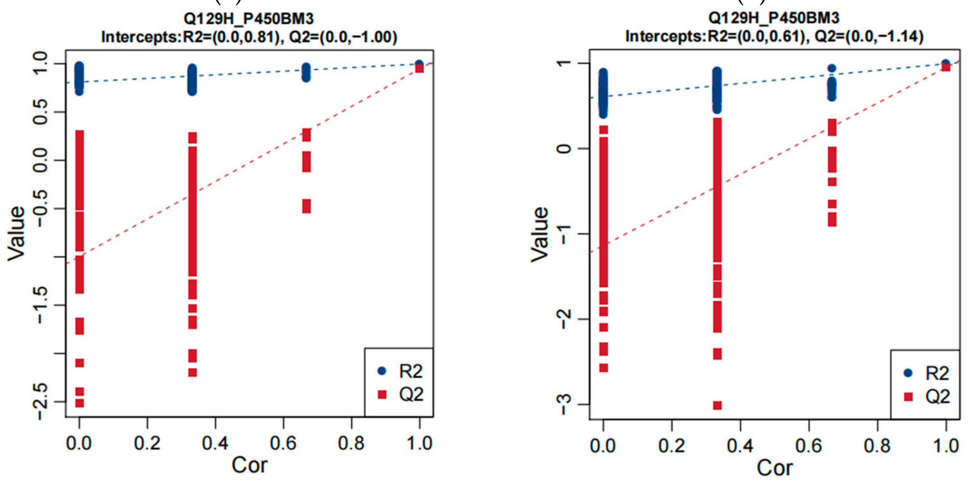

(c) (d)

Figure S5. PLS-DA score scatter plots and permutation validation plots for metabolites in the P450BM3 group and R162H group: (a) Positive ion mode score scatter plot. (b) Negative ion mode score scatter plot. (c) Positive ion mode permutation validation plot. (d) Negative ion mode permutation validation plot.

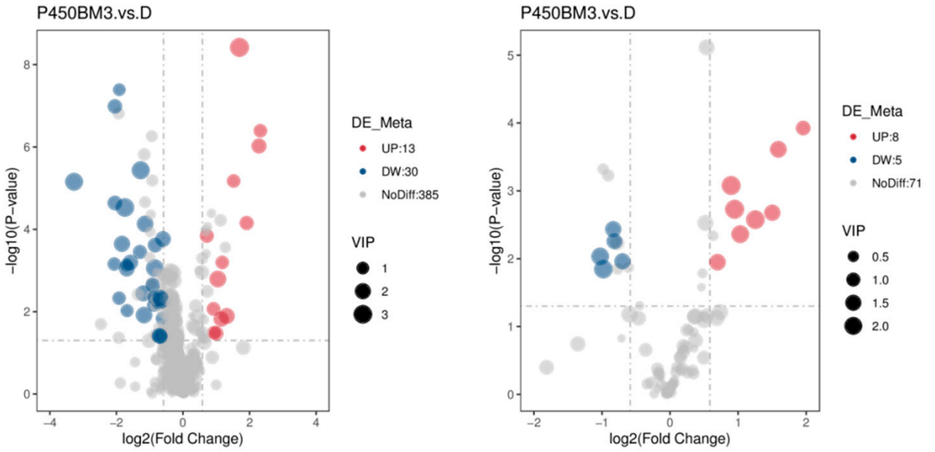

(a) (b)

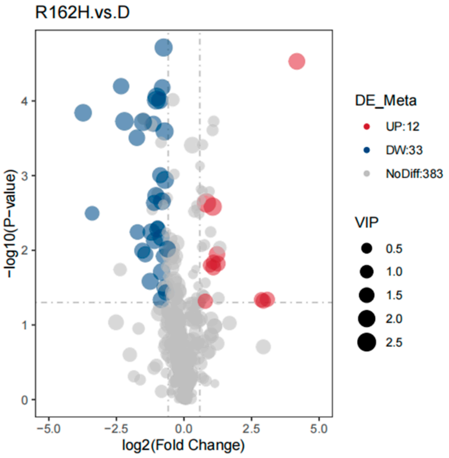
R162H.vs.D
-log10(P-value)
log2(Fold Change)
DE_Meta
UP:12
DW:33
NoDiff:383
VIP
0.5
1.0
1.5
2.0
2.5

(c)

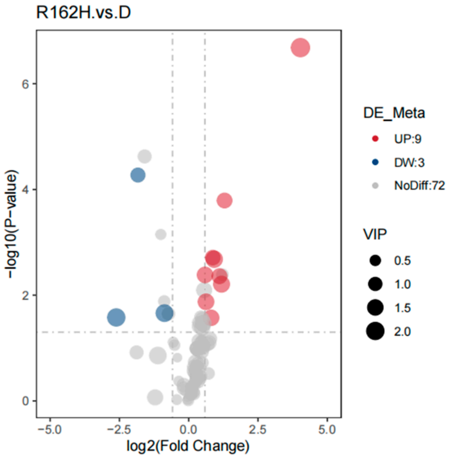
R162H.vs.D
-log10(P-value)
log2(Fold Change)
DE_Meta
UP:9
DW:3
NoDiff:72
VIP
0.5
1.0
1.5
2.0

(d)

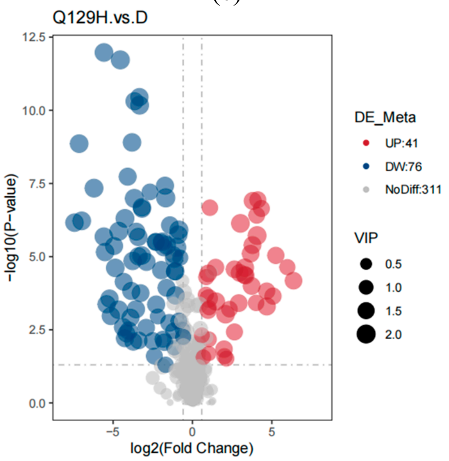
Q129H.vs.D
-log10(P-value)
log2(Fold Change)
DE_Meta
UP:41
DW:76
NoDiff:311
VIP
0.5
1.0
1.5
2.0

(e)

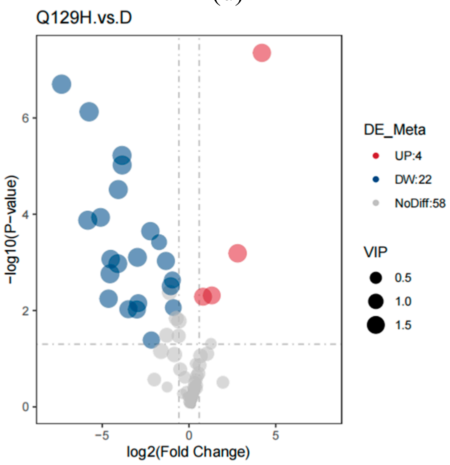
Q129H.vs.D
-log10(P-value)
log2(Fold Change)
DE_Meta
UP:4
DW:22
NoDiff:58
VIP
0.5
1.0
1.5

(f)

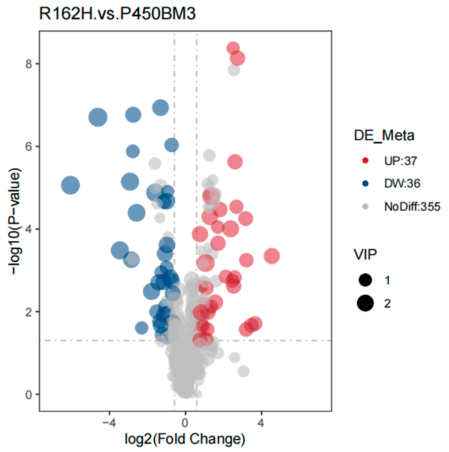
R162H.vs.P450BM3
-log10(P-value)
log2(Fold Change)
DE_Meta
UP:37
DW:36
NoDiff:355
VIP
1
2

(g)

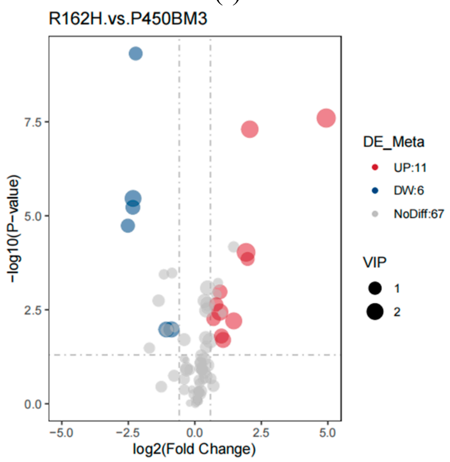
R162H.vs.P450BM3
-log10(P-value)
log2(Fold Change)
DE_Meta
UP:11
DW:6
NoDiff:67
VIP
1
2

(h)

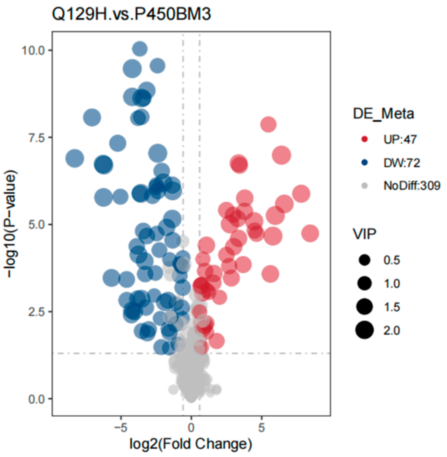

(i)

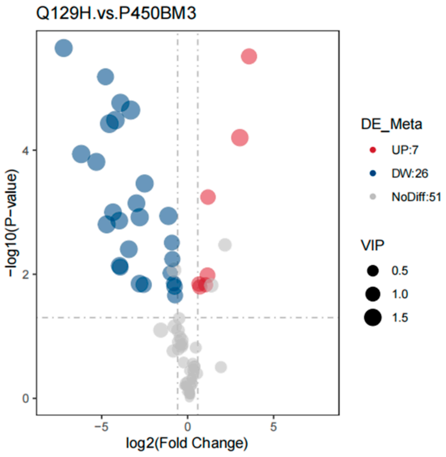

(j)

Figure S6. Volcano plots of differential metabolites: (a) Volcano plots of differentially expressed metabolites in positive ion mode for the P450BM3 group and D group. (b) Volcano plots of differentially expressed metabolites in negative ion mode for the P450BM3 group and D group. (c) Volcano plots of differentially expressed metabolites in negative ion mode for the R162H group and D group. (d) Volcano plots of differentially expressed metabolites in negative ion mode for the R162H group and D group. (e) Volcano plots of differentially expressed metabolites in negative ion mode for the Q129H group and D group. (f) Volcano plots of differentially expressed metabolites in negative ion mode for the Q129H group and D group.(g) Volcano plots of differentially expressed metabolites in positive ion mode for the P450BM3 group and R162H group. (e) Volcano plots of differentially expressed metabolites in negative ion mode for the P450BM3 group and R162H group.(i) Volcano plots of differentially expressed metabolites in positive ion mode for the P450BM3 group and Q129H group. (j) Volcano plots of differentially expressed metabolites in negative ion mode for the P450BM3 group and Q129H group.
